# A novel cytotoxic anti-B7-H3 affibody with therapeutic potential in acute myeloid leukemia

**DOI:** 10.3389/fphar.2025.1684226

**Published:** 2025-09-12

**Authors:** Andrei-Mihai Vasilescu, Adina-Gabriela Vasilescu, Livia Elena Sima, Cristian V. A. Munteanu, Natalia Baran, Ștefan-Eugen Szedlacsek

**Affiliations:** ^1^ Department of Enzymology, Institute of Biochemistry of the Romanian Academy, Bucharest, Romania; ^2^ Department of Molecular Biology of the Cell, Institute of Biochemistry of the Romanian Academy, Bucharest, Romania; ^3^ Department of Bioinformatics and Structural Biochemistry, Institute of Biochemistry of the Romanian Academy, Bucharest, Romania; ^4^ Department of Hematology and Central Hematology Laboratory, Inselspital, Bern University Hospital, University of Bern, Bern, Switzerland; ^5^ Department of Internal Medicine, University of South Dakota, Sioux Falls, SD, United States

**Keywords:** AML, affibody, B7-H3, cytotoxin, magainin-2, leukemia, therapy

## Abstract

**Introduction:**

Acute Myeloid Leukemia (AML) is a group of very aggressive hematological malignancies, with dismal long-term survival rates and little therapeutic recourse presently. The transmembrane ligand B7-H3 is a known therapeutic target, biomarker of response and correlated with an unfavorable prognosis in several malignancies, including AML, due to acquired resistance to immune checkpoint-targeting therapies. Therefore, developing therapeutic strategies with improved efficacy to overcome this obstacle constitutes an unmet need. Our study entails the design, production and *in vitro* testing of a novel recombinant Affibody with high affinity for B7-H3 coupled with the cytotoxic peptide Magainin-2, known for its membranolytic properties and potent antimicrobial and antitumor activity.

**Methods:**

We expressed the conjugate in *Escherichia coli*, affinity purified it and confirmed its sequence by nanoLC-MS/MS. B7-H3-positive AML representative THP-1 cells and B7-H3-negative B-lymphoblastic RAJI cells were used for the experiments. The IC_50_ of the cytotoxic conjugate was determined through MTS assay and its necrotic, apoptotic and antiproliferative activities were evaluated by flow cytometry and Western blot.

**Results:**

Overall, the results show that our cytotoxic anti-B7-H3 affibody possesses strong antiproliferative and cell death-inducing activity that is highly specific to B7-H3-expressing AML cells (IC_50_ against THP-1 cells was 26.35 µM). Moreover, this observed activity of our conjugate is significantly more potent than the previously described activity of Magainin-2 alone.

**Conclusion:**

In conclusion, we designed, produced and evaluated a novel anti-B7-H3 cytotoxic affibody-drug conjugate (AfDC) that, from these preliminary *in vitro* data, shows high potential for translation to the therapy of AML, warranting further preclinical and translational exploration, including pharmacokinetic and *in vivo* efficacy studies.

## 1 Introduction

Acute Myeloid Leukemia (AML) is a group of genetically and morphologically heterogeneous malignant hematological diseases, characterized by the aggressive expansion of immature blast cells in the bone marrow (BM) and peripheral blood. AML frequently displays a short-lived response to intensive standard-of-care chemotherapy and results in a very short overall survival rate due to disease persistence or recurrence (Fan et al., 2023). Compounding these dangerous properties, AML cells frequently exhibit intrinsic or acquired resistance to chemotherapy, often at the level of minimal residual disease, accompanied by metabolic adaptation and immune evasion. By hiding within the BM niche under chemotherapeutic stress, they are capable of developing high resistance to multiple therapies, rewiring and suppressing the immune compartment, thereby increasing the risk of relapse (Wittwer et al., 2017). Given its aggressive nature and high relapse rate, AML represents a grave danger to human life and demands urgent and targeted therapeutic intervention. Thus, developing novel therapeutic strategies for AML remains a critical and unmet medical need.

B7-H3 (CD276) is a transmembrane immune checkpoint ligand protein that belongs to the B7 family and plays a key role in immune regulation. B7-H3, frequently absent or expressed at low levels in physiological conditions, is overexpressed in a wide range of solid tumors (cervical-, colorectal-, and prostate cancer) and some hematologic malignancies, including AML, Myelodysplastic Syndromes (MDS) and others. This rare expression pattern makes B7-H3 an optimal prognostic biomarker for cancer diagnosis and therapy and a predictive biomarker, given that its persisting expression is often associated with poor prognosis (Fan et al., 2023; He et al., 2025; Tolmac et al., 2025). Mechanistically, B7-H3 is involved in the inhibition of the immune response by modulating and regulating T_reg_ cells and NK cells in particular (Guo et al., 2025). Notably, although the primary roles of B7-H3 have been described, the precise mechanism underlying its interaction with specific receptors remains incompletely understood and requires further investigation (Tan and Zhao, 2024).

In cancer, high B7-H3 expression acts as a protective mechanism, because it inhibits immune system effectors and increases proliferation, growth and survival of tumor cells through its downstream signaling pathways (Guo et al., 2025). Its abundant expression and involvement in immune evasion make B7-H3 a promising therapeutic target for innovative approaches aiming far beyond monoclonal antibodies, Chimeric Antigen Receptor (CAR)-T therapy, or bispecific antibodies, to combine direct anti-leukemic effects with reversing immunosuppression activities, represented by a new generation of AfDC (Affibody-Drug Conjugates).

Affibodies are highly effective targeting proteins, with a simple three alpha-helical structure and high affinity for their epitopes. These are highly soluble, resistant to thermal changes and protease activity and can be rapidly distributed into the bloodstream thanks to their very small size of ∼6–7 kDa. Their size also affords them rapid clearance from the organism, an essential aspect for therapy (Hö gbom et al., 2003; Oroujeni et al., 2023). Oroujeni et al. previously matured, extensively characterized and validated a highly promising amino acid sequence of the affibody molecule SYNT-179. By using a solid methodology, they demonstrated high specificity of this affibody towards B7-H3 *in vitro* (radioactive labeling) and *in vivo* (nanoSPECT/CT imaging on xenografted mice) and an excellent affinity of *K*_*D1*_ = 0.028 ± 0.001 nM for the rarest epitope of B7-H3 and *K*_*D2*_ = 8.2 ± 0.5 nM for the most abundant epitope, as measured by LigandTracer (Oroujeni et al., 2023). The same group recently proved in a tumor xenograft model of ovarian cancer that SYNT-179 is an excellent candidate with clinical potential for imaging diagnostics of B7-H3-expressing cancers (Tolmac et al., 2025).

On the search for natural peptides with anticancer properties, recent studies showed that the Magainin class of natural peptides isolated from the skin of the frog *Xenopus laevis* are not only potent antibacterial agents (Zasloff, 1987), but are also very effective against a wide range of both solid and liquid tumor cells (Baker et al., 1993). Of these isolated peptides, Magainin-2 (MAG2) has emerged as the most promising peptide due to its superior anti-cancer potency, as eloquently demonstrated *in vitro* by Cruciani et al. (1991) and later by Ohsaki et al. (1992). Mechanistically, electron microscopy studies have demonstrated that MAG2 exerts its cytotoxic effects by disrupting the integrity of the cell membrane through pore formation, followed by an efflux of essential cytosolic ions, enzymes, nutrients and other critical components. Ultimately, MAG2 leads to destabilization of all cellular functions and necrotic cell death (Lehmann et al., 2006).

We hypothesized that coupling the high-affinity anti-B7-H3 affibody with the membrane-disruptive peptide MAG2 would synergistically enhance the antileukemic effect by selectively targeting AML cells. Affibody-mediated binding would position MAG2 close to the cell membrane, promoting its insertion and progressively destabilizing the lipid bilayer. Upon reaching a critical threshold of MAG2 accumulation, membrane integrity would collapse, leading to rapid and irreversible cell death.

In this study, we present a novel, targeted cytotoxic protein construct, Aff-MAG2, which combines the most advanced anti-B7-H3 affibody available, SYNT-179, with the potent membrane-lytic peptide MAG2. This recombinant fusion protein is designed to selectively and synergistically target the promising AML biomarker B7-H3, aiming to induce cancer cell death with minimal off-target toxicity.

We describe the design and production of Aff-MAG2, involving genetic fusion of the matured anti-B7-H3 affibody with the cytolytic peptide MAG2, followed by expression of the recombinant construct in *Escherichia coli* and its subsequent purification. We next aimed to elucidate the cytotoxic mechanism of action by evaluating the construct’s effects on viability, proliferation and cell death through MTS assay, flow cytometry, and Western blotting.

To our knowledge, this is the first report of a B7-H3-specific affibody-drug conjugate incorporating MAG2 for targeted membrane disruption and selective anti-leukemic activity, warranting further preclinical and translational studies.

## 2 Materials and methods

### 2.1 Expression plasmid

We ordered the pET-19b expression plasmid vector from GenScript with the DNA sequence of the 6xHis-SUMO-Aff-MAG2 construct inserted between the unique restriction sites NcoI at 5′ and XhoI at 3’. The plasmid was provided in lyophilized form and it was resuspended upon arrival in TE Buffer (Supplementary Table S2) at a final DNA concentration of 200 ng/μL.

### 2.2 Cell lines

The cell lines utilized were THP-1 (Acute monocytic leukemia monocytes), as a representative cell line for AML, from the American Type Culture Collection (ATCC, TIB-202) and RAJI (Burkitt lymphoma B-lymphocytes, ATCC, CCL-86) (ATCC, 2025), as the negative control, which were a kind gift from Dr. Adrian Bogdan Țigu from the Research Centre for Advanced Medicine–MEDFUTURE at Iuliu Hațieganu University of Medicine and Pharmacy, Cluj-Napoca, Romania. Both cell lines were Mycoplasma-free and were used for experiments at passages between 3 and 9. All cells were grown in suspension in RPMI 1640 cell culture medium with L-Glutamine (PanBiotech, product no. P04-16500) supplemented with 10% inactivated fetal bovine serum (FBS), MEM Non-Essential Amino Acids Solution (Gibco, ref. no. 11140-050) and a cocktail of 50 I.U./mL penicillin and 50 μg/mL streptomycin at 37 °C, in a 5% CO_2_ humidified atmosphere. All cell cultures were passaged regularly and maintained at the densities recommended by the ATCC (ATCC, 2025). As an exception, during all treatments with the test compound, all cells were resuspended in the described medium without added antibiotics. For all cell line experiments in this study, THP-1 cells were seeded at a density of 1 × 10^5^ viable cells/mL, while RAJI cells were seeded at 5 × 10^5^ viable cells/mL, unless otherwise specified.

### 2.3 Expression and purification of 6xhis-SUMO-Aff-MAG2

For the expression of the recombinant fusion protein, we utilized the BL21 (DE3) *E. coli* strain, as it is optimal for the pET-19b expression vector. First, we transformed 50 µL suspension of super competent bacteria with 200 ng of the expression plasmid, utilizing the heat shock method (10 min on ice, followed by 20 s at 42 °C, then 2 min on ice). The transformed cells were plated on a 100 µg/mL ampicillin in solid Luria-Bertani (LB)-agar medium for selection of colonies that incorporated the plasmid and incubated 16 h at 37 °C. Then, we selected a medium-sized colony and introduced it in liquid LB growth medium supplemented with 100 μg/mL ampicillin for selection, constituting the seeding culture. We grew the seeding culture at 20 °C, at 130 RPM for 17 h, so as to maintain it in the exponential growth phase until the next day. Then, the seeding culture was grown for another 3 h at 37 °C, 140 RPM, until its OD_600_ reached ∼0.7. For the main culture, we utilized 1% seeding culture diluted in LB medium supplemented with 100 μg/mL ampicillin for selection. This culture was grown for 2.5 h at 37 °C, 120 RPM, then at a temperature decreasing gradually from 37 °C to 18 °C over the course of 30 min, until an OD_600_ of ∼0.7 was reached. We employed this temperature ramp to prepare the culture for the protein expression induction conditions of 18 °C, 120 RPM, for 15 h. Induction was initiated by adding isopropyl β-D-1-thiogalactopyranoside (IPTG) to the culture, up to a final concentration of 0.5 mM. After measuring OD_600_ of the overnight induced culture, we collected the bacteria by centrifugation at 8,000 xg, for 10 min at 4 °C and stored the resulting pellet at −80 °C. SDS-PAGE samples were prepared from the grown uninduced and induced cultures, proportionally to their OD_600_, by adding Laemmli buffer over the bacterial pellets, resuspending, boiling at 95 °C for 5 min and then centrifuging for 2 min at 12,000 xg. All SDS-PAGE samples utilized in this study were prepared the same way.

For bacterial lysis, we resuspended the pellet in 10 mL Bacterial Lysis Buffer (Supplementary Table S2) *per* G cell wet weight. For determining cell wet weight, we utilized a rule of thumb of 1.7 g cell wet weight *per* L of culture *per* unit of OD_600_ (Glazyrina et al., 2010). For the solubility test, lysis was done through sonication on a Bandelin Sonopuls HD 2070, at 50% power, for 5 cycles of 30 s pulse – 30 s pause each, in an ice bath. For protein purification purposes, we lysed bacteria by running it three times through a French Press Cell Disrupter (Thermo), on a high ratio and 1000 PSI. The lysate was then centrifuged at 14,000 xg for 40 min at 4 °C. Electrophoresis samples were prepared from both the lysate pellet and supernatant for evaluation. After collecting the lysate supernatant, we filtered it through a 0.45 µm pore diameter filter (Santa Cruz, cat. no. sc-358818).

For purification of 6xHis-SUMO-Aff-MAG2 from the bacterial lysate, we utilized an ÄKTAprime plus FPLC (G&E Healthcare) and a HisTrap HP 5 mL Ni-Sepharose affinity chromatography column (Cytiva, lot no. 10330203) to bind the 6xHis tag of the recombinant protein. All purification operations were performed in a 4 °C environment. All purification buffers were initially filtered as described before and then degassed. The purification method was the following: 10 column volumes (CV) MilliQ water, followed by column equilibration with 10 CV Binding Buffer (Supplementary Table S2), lysate loading on the column through the superloop, followed by washing with Binding Buffer for 15 CV, then fractionation in a 0%–100% gradient of Elution Buffer (Supplementary Table S2) over 50 mL. The fractions were 1 mL each. All the column equilibration and washing steps were done at a flow rate of 5 mL/min, while the lysate loading and the fractionation steps were done at 1 mL/min. Samples of insoluble and soluble proteins from the bacterial lysate, relevant eluted protein fractions and of column flowthrough were prepared for SDS-PAGE analysis. All of the SDS-PAGE gels used for protein evaluation in this study were 15% Tris-glycine gels, made in-house, run in SDS-PAGE Tank Buffer, at 30 mA, for 50 min and stained with Coomassie Brilliant blue G 250, unless otherwise stated. The molecular weight markers utilized were PageRuler Prestained and PageRuler Plus Prestained Protein Ladders (Thermo Fisher Scientific). The eluted fractions evaluation gel was 18%. We dialysed reunited eluted protein fractions utilizing Snakeskin Dialysis Tubing with a MWCO of 3.5 kDa (Thermo, ref. no. 88242) in Dialysis Buffer (Supplementary Table S2), in one step of 16 h, followed by another step of 4 h at 4 °C. After dialysis, we determined purified 6xHis-SUMO-Aff-MAG2 concentration utilizing the Pierce 660 assay (Thermo Scientific, cat. no. 22660) with Dialysis Buffer as blank.

We performed cleavage of all recombinant construct tags in the protein’s buffer (Dialysis Buffer), using a 6xHis-SUMO protease (Ulp1 protease from *S. cerevisiae*) produced in-house, at a 20:1 recombinant protein:SUMO protease mass ratio, for 19 h at 4 °C. Then, the tags and the SUMO protease were bound to pre-equilibrated Pure Cube Indigo Ni-Agarose resin (Cube Biotech, cat. no. 75103) for 17 h with rotation, at 4 °C. After a 500 xg, 3-min centrifugation at 4 °C, the pure Aff-MAG2 was separated in the supernatant and collected. SDS-PAGE samples were prepared to evaluate tag cleavage, separation and final cytotoxic protein purity. The purity of the final compound was quantified through band densitometry, utilizing ImageJ (Schneider et al., 2012). Finally, Aff-MAG2 was lyophilized in a Martin Christ Lyophilizer at −56 °C, for 16 h, followed by resuspension in sterile MilliQ water to a final cytotoxic compound stock concentration of 1 mM, for use in cell-based experiments.

### 2.4 NanoLC-MS/MS analysis of the purified Aff-MAG2 construct and evaluation of the hypothetical cleavage site

We used the purified protein sample in Dialysis Buffer (Supplementary Table S2) for these experiments, employing an in-solution trypsin digestion protocol. Following protein denaturation in 8 M urea in 100 mM Tris-HCl, Cys disulfide bridges were reduced by sample incubation with 10 mM DTT in 50 mM ammonium bicarbonate (ABC) for 1 hour at room temperature (RT, 25 °C). After reduction, the sample was alkylated in 55 mM iodoacetamide in 50 mM ABC for 1 hour at RT. This excess alkylating agent was inactivated with 10 mM DTT, by adding two times the volume of the alkylating buffer used, for 1 hour at RT. Then, urea in the sample was diluted to <1.0 M with 50 mM ABC and the pH was adjusted to approximately 7.7 with 50 mM ABC, for optimal trypsin digestion. Trypsin digestion was performed by adding trypsin buffer (100 ng/μL sequencing grade trypsin from Promega, cat. no. V5111 in 50 mM acetic acid) until the w:w ratio of trypsin:protein in the sample was 1:50 and then incubating the sample for 16 h at 37 °C. We then desalted the obtained peptides as previously described (Munteanu et al., 2021). Subsequently, the sample was concentrated to dryness and kept at −20 °C until injection.

For nanoLC-MS/MS analysis, an Easy-nLC 1200 coupled to an LTQ Orbitrap™ Velos Pro (Thermo Fisher Scientific) mass spectrometer was used. The sample was reconstituted in solvent A (0.06% formic acid - FA and 2% acetonitrile - ACN) and the peptides were separated on an RP C18 Acclaim™ PepMap™ 100 (0.075 um x 150 mm) analytical column (Thermo Fisher Scientific) using a 2%–30% solvent B (0.06% FA and 80% ACN) for ∼90 min. The data acquisition involved a data-dependent procedure under which the top five most abundant ions from the survey scan acquired in the orbital trap were selected for fragmentation using either CID or HCD, alternatively, in different runs, as previously described (Chirițoiu et al., 2021). The data were searched against the *E. coli* proteome (including subtaxonomies) to which we added the recombinant sequence using SequestHT under Proteome Discoverer v2.5 environment (Thermo Fisher Scientific) with the following settings: enzyme Trypsin (with semi-tryptic specificity), 10 ppm maximum allowed mass deviation for precursors, 0.5 Da for the fragments mass tolerance acquired in the linear trap, 0.02 Da for MS/MS ions acquired in the Orbitrap, Met oxidation as a variable modification and Cys Carbamidomethylation as a fixed modification. The Target/Decoy strategy was used to filter only PSMs at 1% FDR.

### 2.5 MTS assay

Cells were plated in 96-well plates, with both THP-1 and RAJI cells necessary for all experimental conditions, incubated for 12 h before treatment. For every experimental condition, we performed a technical triplicate. Both cell lines were treated with the following concentrations of Aff-MAG2: 1.56 µM, 3.12 µM, 6.25 µM, 12.5 µM, 25 μM, 50 μM and 100 µM. After the treatment incubation of 6 h, the MTS reagent - CellTiter 96^®^ AQ_ueous_ One Solution Reagent (Promega, ref. no. G358A) was added directly to the wells, proportionally to the final solution volume and incubated at 37 °C. The absorbance at 490 nm of the colored reaction product was read after shaking at FLUOstar^®^ Omega (BMG Labtech), every 15 min, until fully developed. The blank was the complete medium without antibiotics the cells were seeded in, plus MTS reagent.

### 2.6 Flow cytometry

For determination of cell surface expression of B7-H3, 1 × 10^5^ THP-1 or RAJI cells, respectively, were used for each experimental condition. After we transferred the necessary cell suspension into FACS tubes, we centrifuged them at 350 xg, for 5 min at 4 °C, washed them with FACS Buffer (Supplementary Table S2) and resuspended them in 100 µL of the same buffer, to a corresponding final density of 1 × 10^6^ cells/mL. Afterwards, cells were incubated with PE/Cyanine7 anti-human CD276 (B7-H3) mouse monoclonal Antibody (BioLegend, #351007) and PE/Cyanine7 mouse monoclonal Isotype Ctrl Antibody (BioLegend, #400125), respectively, for 20 min on ice, in the dark. The primary anti-B7-H3 antibody and the Isotype control were of the same species, type and chain and were used at identical final concentrations. After washing the cells two times with FACS Buffer and resuspending them in 100 µL of the same buffer, we acquired 10,000 events for each experimental condition utilizing the 488 nm laser and corresponding PE-Cy7 detection filter. For all flow cytometry experiments, we utilized the BD FACSVerse^™^ instrument (BD Biosciences) for acquisition. For the B7-H3 expression experiment, the Isotype control was utilized to determine baseline fluorescence.

For the necrosis and apoptosis experiment, we seeded the cells at 1 × 10^6^ cells/mL in 12-well plates, left them for 12 h and then treated both THP-1 and RAJI cells with 30 µM Aff-MAG2 for 6 h. After incubation and a centrifugation at 350 xg, for 5 min at 4 °C, we resuspended the cells in Annexin Buffer (Supplementary Table S2), counted them with a hemocytometer and then diluted them to 1 × 10^6^ cells/mL with Annexin Buffer. Then, 100 µL of cells were incubated for 15 min at RT in the dark with 5 µL of FITC Annexin V (BioLegend, #640905), 5 μg/mL Propidium Iodide–PI (BioLegend, #421301), or both. For both cell lines, the negative controls were unstained, untreated cells. The single-stain positive controls used for compensation were cells treated 18 h with 30 µM Cisplatin for FITC Annexin V brightest signal and 15 min with Triton X-100 0.02% for PI single-stained control, respectively. After diluting the samples with 400 µL Annexin Buffer, we acquired 10,000 events for each sample using the 488 nm laser and corresponding FITC and PI filters. For optimum data acquisition, the unstained cells were used to determine autofluorescence and the single-stain controls were used for the automatic calculation of the compensation matrix of the bleed-through effects between the FITC and PI fluorescent signals.

In the Cytobank platform (Kotecha et al., 2010), we analyzed the flow cytometry data, starting with generating the compensation matrix and designing the gating strategies. The first applied gate was on an FSC-A vs. SSC-A dot plot to select intact cells (only applied in the B7-H3 expression experiment), the second gate was on an FSC-A vs. FSC-H dot plot, to select only singlet cells and the third gate delimited viable cells from necrotic and apoptotic cells, respectively, on a FITC vs. PI fluorescence dot plot.

### 2.7 Western blot

For the Western blot experiments, we seeded the cells in 6-well plates, incubated them for 12 h and then treated THP-1 and RAJI cells with 15 µM Aff-MAG2 for 6 h. After treatment, the cells were washed with PBS and pelleted at 350 xg, for 5 min at RT and then frozen at −80 °C. They were then lysed by rapid thawing, resuspension in Eukaryote Lysis Buffer (Supplementary Table S2), pushed through a sterile syringe needle 5 times and shaken for 40 min at 4 °C. After shaking, we centrifuged the lysate at 20,000 xg for 40 min at 4 °C and then collected the supernatant. We employed the Pierce^™^ BCA Protein Assay Kit (Thermo Fisher, cat. no. 23225) to determine total protein concentration and after 30 min at 37 °C, the absorbance was measured at 562 nm at FLUOstar^®^ Omega, with the Eukaryote Lysis Buffer as blank. Then, the lysate was prepared for SDS-PAGE, as already described. Either made-in-house Tris-glycine gels, or pre-cast gels (Bio-Rad) were utilized as appropriate for proper separation of the bands of interest. After electrophoresis, the proteins were transferred to PVDF membranes in a cold wet system, for 1 h and 30 min, at 350 mA, in transfer buffer with 20% ethanol. The membranes were then blocked for 1 h at RT in 5% non-fat milk in Tris-buffered saline-Tween (TBS-T) solution and incubated with primary antibodies for 16 h, at 4 °C. The following primary antibodies were used at indicated dilutions in blocking solution: anti-CD276 (B7-H3), mouse monoclonal (BioLegend, #616951), 1:2000 dilution (incubated 1 h at RT); anti-Ki-67, mouse monoclonal (BioLegend, #350501), 1:1000 dilution; anti-cleaved caspase 3, rabbit monoclonal (Cell Signaling, #9661S), 1:1000; anti-alpha tubulin, rabbit polyclonal (Abcam, #ab15246), 1:8000. All membrane washes were conducted at RT in TBS-T 3 times, for 5 min after primary antibodies and 10 min after secondary antibodies. After washing, the following secondary horseradish peroxidase (HRP)-conjugated antibodies were applied for 1 h at RT, at a 1:4000 dilution in blocking solution, as appropriate for the species of the primary antibody to be detected: HRP-goat anti-mouse polyclonal (BioLegend, #405306) and HRP-mouse anti-rabbit polyclonal (Invitrogen, #61-6520). The signal detection was done either with Immobilon^®^ Crescendo Western HRP substrate (Merck, #WBLUR0100), or SuperSignal^™^ West Femto Maximum Sensitivity Substrate (Thermo Fisher, #34094) and the images were acquired at a ChemiDoc^™^ MP Imaging System (Bio-Rad).

### 2.8 Data analysis and statistics

We performed all experiments independently three times and we employed at least two biological replicates. We fitted and plotted the inhibitory dose-response curves and calculated the IC_50_ of Aff-MAG2 in GraphPad Prism v9.3.0 (Dotmatics). To determine statistical significance, we utilized an Unpaired one-tailed *t*-test in GraphPad Prism v9.3.0. *p* < 0.05 was considered statistically significant.

## 3 Results

### 3.1 Design and development of a novel recombinant affibody targeting B7-H3 fused to magainin-2

**FIGURE 1:**
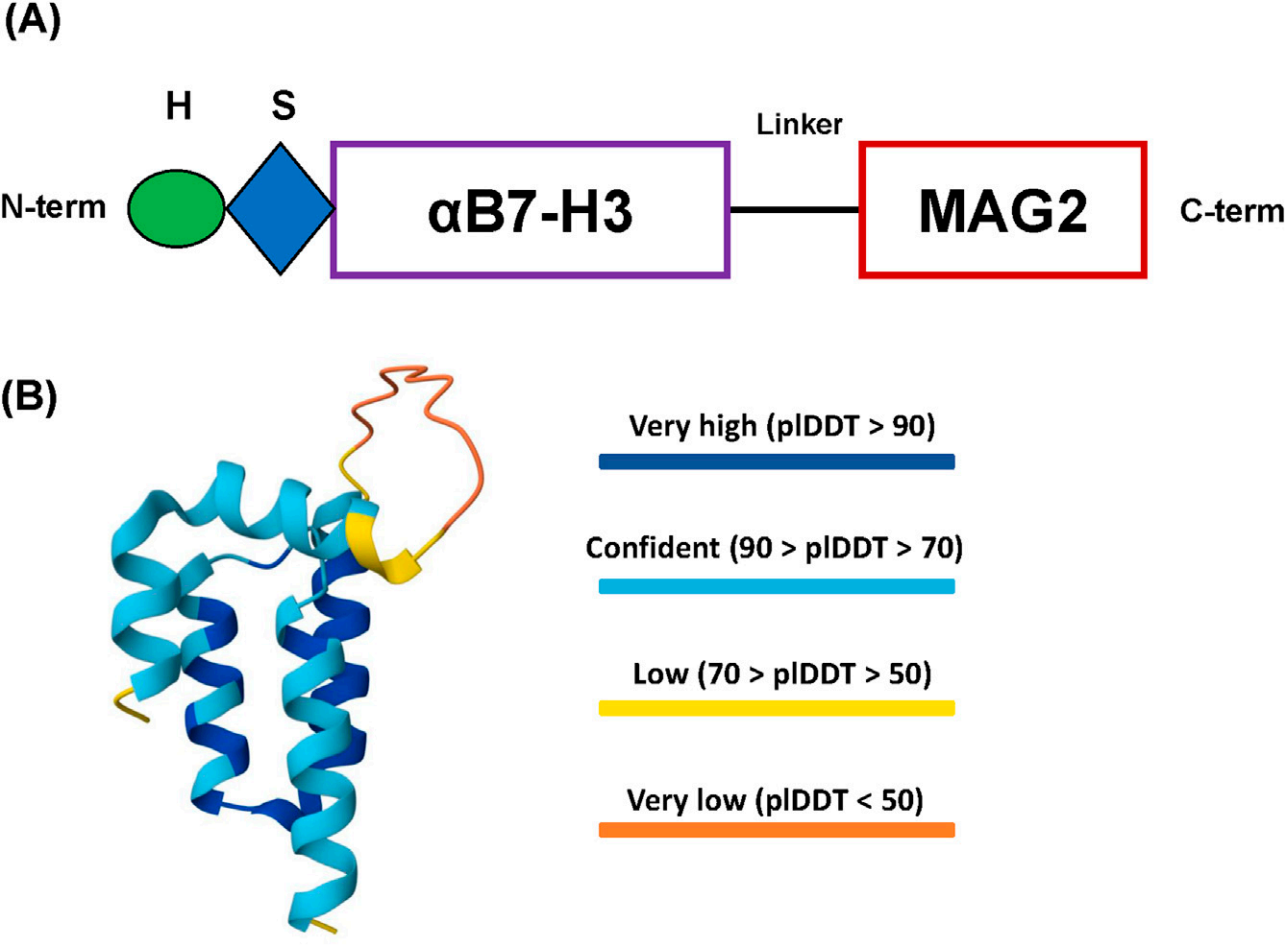
Design of the recombinant protein construct. **(A)** Schema of the 6xHis-SUMO-Aff-MAG2 recombinant protein construct. H, 6xHis tag; S, SUMO tag; αB7-H3, Anti-B7-H3 affibody; MAG2, Magainin-2. **(B)** AlphaFold 3 model of the final recombinant protein structure. The N-terminus and the C-terminus are coincidentally the yellow parts at the end of two of the alpha-helices. The protein is most likely ordered into 3 successive alpha-helices comprising the affibody, followed by the disordered linker region and ending with the alpha-helix of Magainin-2. plDDT - predicted local Distance Difference Test.

First, we designed the amino acid sequence of the fusion construct according to established guidelines of recombinant protein rational design (Li, 2011; Zorko et al., 2010; Chen et al., 2013). We used SYNT-179 as the affibody molecule for our construct, followed by a triple GGGGS linker towards the C-terminus and ending with the toxin Magainin-2 sequence. For affinity purification, we added a 6xHis tag at the N-terminus, followed by a Small Ubiquitin-related MOdifier (SUMO) tag (SMT3 from *S. cerevisiae*) for separating the tags from the final recombinant protein after purification (Figure 1A). The amino acid sequence of the affibody (SYNT-179), from N-term to C-term, is: AEAKFAKEKINALGEIIWLPNLTYDQIKAFIAKLNDDPSQSSELLSEAKKLSESQ, while for MAG2 it is: GIGKFLHSAKKFGKAFVGEIMNS. We selected the (GGGGS)_3_ linker due to its established stability, flexibility and resistance to proteolytic degradation. Its repeated sequence provides sufficient length to minimize steric hindrance between the functional domains of the construct while preserving the conformational freedom necessary for their independent activity (Chen et al., 2013). The SUMO tag accomplishes several essential roles in the design of our protein construct. First, it enables precise cleavage by the SUMO-specific protease Ulp1 (from *S. cerevisiae*), which only recognizes the tertiary structure of the SUMO tag and cleaves immediately downstream of it, without leaving residual amino acids on the N-terminus of the protein of interest (Li, 2011). This is especially important for targeting molecules like the affibody, which often require a native N-terminus for proper folding and function. Second, the SUMO tag protects the construct from proteolytic degradation and reduces or shields against the antibacterial activity of Magainin-2 during expression in *E. coli*, likely due to electrostatic interactions between the negatively charged SUMO tag and the cationic peptide at physiological pH (Bommarius et al., 2010). Finally, the affibody, linker, toxic peptide and the SUMO tag display high aqueous solubility, which facilitates the construct’s efficient recovery from the bacterial lysate supernatant in high yields (Tolmac et al., 2025; Zasloff, 1987; Li, 2011; Chen et al., 2013).

The structure of the Aff-MAG2 fusion protein was modeled using AlphaFold 3 Server (Abr et al., 2024). The predicted structure revealed stable alpha-helical conformations of both the anti-B7-H3 affibody and MAG2, as shown in Figure 1B, with high model confidence scores across the entire construct. Of note, the model suggests a potential interaction between the affibody and the toxic peptide, implicating the possible interactions and functionality of the construct. In parallel, other key physico-chemical properties of the construct necessary for downstream optimization and the purification procedure were calculated and predicted utilizing ProtParam from Expasy (pI, molecular weight, protease cleavage sites) (Gasteiger et al., 2005) and SCRATCH Protein Predictor (SOLpro - solubility upon overexpression in *E. coli*), respectively (Cheng et al., 2005).

### 3.2 Expression and purification of Aff-MAG2 from *Escherichia coli*

**FIGURE 2:**
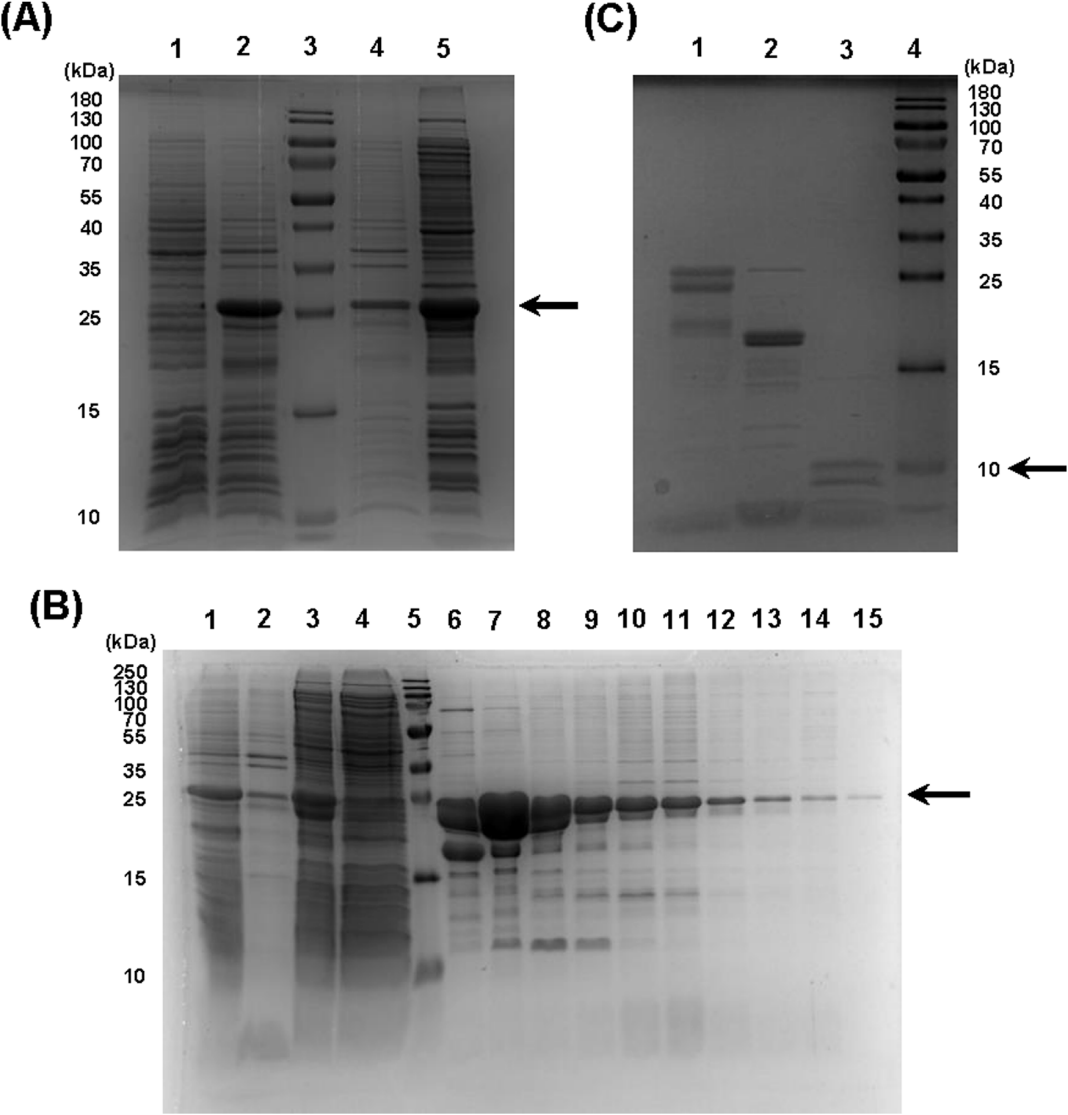
Expression and purification of the recombinant protein. **(A)** 6xHis-SUMO-Aff-MAG2 expression and solubility. 1, bacterial culture not induced; 2, induced culture; 3, molecular weight marker; 4, insoluble fraction from lysed induced bacteria; 5, soluble fraction from lysed induced bacteria. The arrow indicates the recombinant protein. **(B)** 6xHis-SUMO-Aff-MAG2 purification. 1, induced culture; 2, insoluble fraction of lysate; 3, lysate supernatant; 4 - purification column flowthrough; 5, molecular weight marker; 6–15, eluate fractions in the range 35–67, as seen on chromatogram. The arrow indicates the recombinant protein. **(C)** Recombinant protein tag cleavage and separation. 1, negative control reaction without Ulp1; 2, protein bound to resin; 3, supernatant with unbound protein; 4, molecular weight marker. The arrow indicates untagged Aff-MAG2.

Owing to the efficiency of our rational construct design, we successfully expressed the recombinant protein in *E. coli* utilizing a prokaryotic expression vector (Supplementary Figure S1). By employing a reduced expression temperature of 18 °C to promote proper protein folding, the majority of the expressed product was recovered in the soluble fraction (Figure 2A, lane 5). Subsequently, utilizing Immobilized Metal Affinity Chromatography (IMAC), we obtained relatively pure fractions of our protein (Figure 2B, lanes 6-15; Supplementary Figure S2), with effective binding to the Ni-Sepharose column (Figure 2B, lane 4 - the column flowthrough). After dialysis, the combined protein fractions were submitted to the last step of purification. We cleaved the tags with SUMO protease and then separated the final Aff-MAG2 untagged compound in the supernatant using Ni-Agarose resin. However, we observed some degradation of the recombinant protein during the purification step. Nonetheless, there was clear evidence that both the tag cleavage and resin binding were >95% effective (Figure 2C). Although the recombinant protein degraded during purification, this final step of separation increased the purity of the protein. Taken together, the two clear bands in the supernatant (Figure 2C, lane 3) represent the recombinant final protein with a purity of >95%. Most contaminating proteins were retained in the bound fraction (lane 2). The final yield of >95% pure Aff-MAG2 we obtained was ∼12 mg/L of bacterial culture, a satisfying yield, given the fact that the final untagged protein has a molecular weight 2.4 times lower than the expressed full-length construct.

### 3.3 Amino acid sequence confirmation of the purified Aff-MAG2 by nanoLC-MS/MS analysis

**FIGURE 3:**
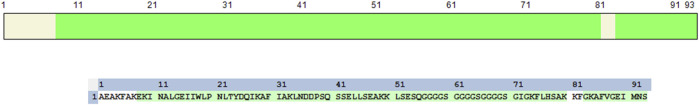
Validation of the purified Aff-MAG2 amino acid sequence. Sequence coverage of the purified recombinant protein. The figure displays the amino acid sequence of the purified construct and highlighted in green is the obtained sequence coverage from the nanoLC-MS/MS analysis.

By using a bottom-up approach employing in-solution trypsinization, we validated the amino acid sequence of Aff-MAG2 by mass spectrometry analysis. Semi-tryptic analysis of the LC-MS/MS data revealed the identification of 66 peptides yielding a total sequence coverage of 88.17% (Figure 3), confirming the identity of the protein we purified and its suitability for downstream cell-based experiments.

### 3.4 Aff-MAG2 demonstrates an inhibitory effect on acute myeloid leukemia cells

**FIGURE 4:**
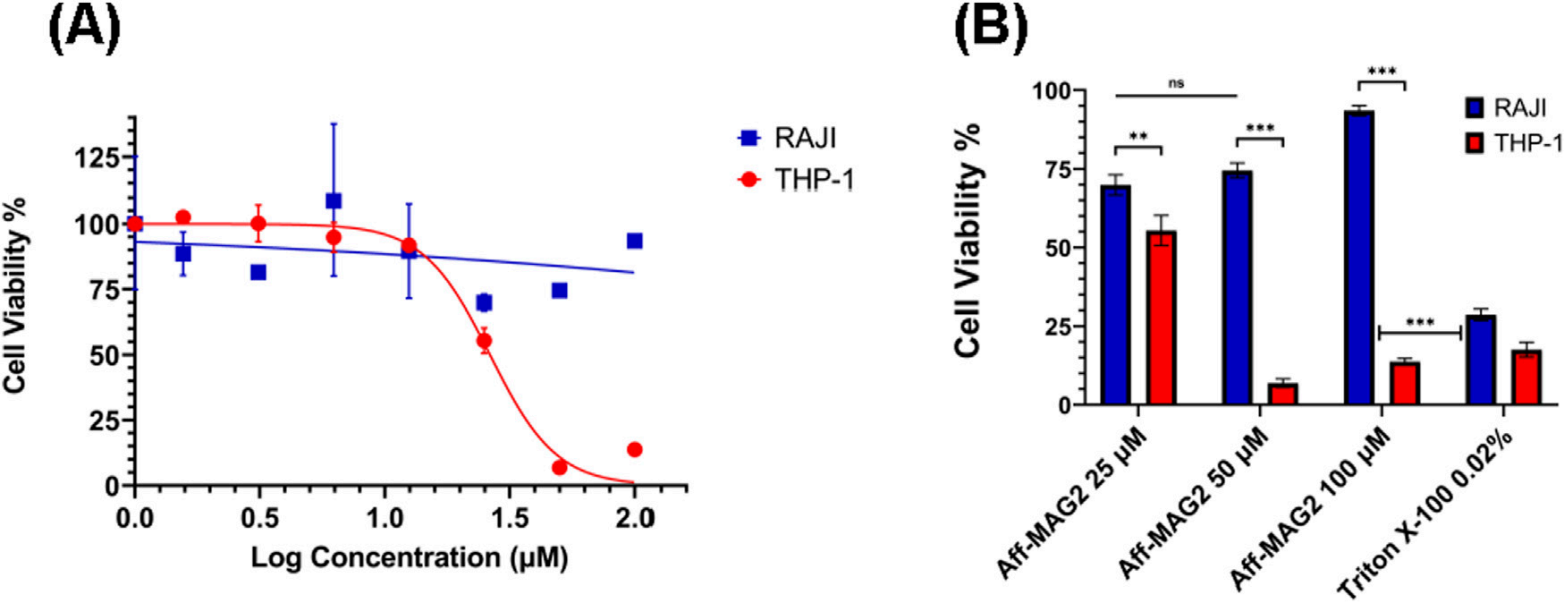
Specific inhibition effect on AML cells in MTS assay. THP-1 (B7-H3^+^) and RAJI (B7-H3^-^) cells were treated with Aff-MAG2 for 6 h. **(A)** Inhibitory dose-response curves. The points on the curves represent, in order from left to right, mean percentage cell viability, normalized to untreated cells, at the following concentrations of cytotoxic compound: 0 μM, 1.56 µM, 3.12 µM, 6.25 µM, 12.5 µM, 25 μM, 50 μM and 100 µM. **(B)** MTS assay-derived pairwise comparison analysis between mean percent viability of RAJI and THP-1 cells, respectively, normalized to untreated cells, for treatment with 25 μM, 50 μM and 100 µM of Aff-MAG2, respectively. Triton X-100 0.02% - positive control for membrane permeabilization. Error bars represent ±SD, *n* = 3. Unpaired one-tailed *t*-test, ns–not significant (*p* ≥ 0.05), **p < 0.01, ***p < 0.001. Data show results from a representative experiment out of three performed.

After purifying and validating our recombinant cytotoxic compound, we aimed to test its potential for inhibiting the viability of AML cells in a specific, targeted way, given its affibody part with high affinity for B7-H3. As a classical model for AML, we chose the THP-1 cell line, known to express B7-H3. To compare the on-target activity of the construct, in parallel, we tested the lymphoblastic RAJI cells, known to lack B7-H3 expression. To this end, we treated both THP-1 and RAJI cells with a serial dilution of Aff-MAG2, at the final concentration range: 0 μM, 1.56 µM, 3.12 µM, 6.25 µM, 12.5 µM, 25 μM, 50 μM and 100 µM. All treatments were performed for 6 h, in technical triplicate, followed by MTS assay. As mentioned above, RAJI cells represented the negative control for specificity of the cytotoxic product. The technical negative controls for the assay comprised of cells from both lines incubated with only the complete growth medium, without added antibiotics (untreated cells). As the hypothesized mechanism of action of the tested compound is the permeabilization of the cell membrane, even leading to pore formation, the technical positive control we utilized for the assay was a 15-min treatment with Triton X-100 0.02%. As Figure 4A shows, THP-1 cell growth was inhibited upon treatment with Aff-MAG2 in a dose-dependent manner. On the other hand, RAJI cells maintained their viability upon treatment with all the tested concentrations of cytotoxin. For example, the mean percent viability difference between the 25 μM and 50 µM treatments was not statistically significant for RAJI (*p* > 0.05, Figure 4B). In contrast, the nonspecific Triton X-100 caused strong inhibition of RAJI cells, even when compared to the 100 µM Aff-MAG2 treatment (*p* < 0.001, Figure 4B), strengthening the hypothesis that the effect of our compound is specific to B7-H3^+^ cells. The difference in the inhibition effect between RAJI and THP-1 was statistically significant for the 25 µM treatment (*p* < 0.01) and highly significant for the 50 μM and 100 µM treatments (*p* < 0.001), as shown in Figure 4B. The calculated half-maximal inhibitory concentration (IC_50_) of Aff-MAG2 against THP-1 (B7-H3^+^) cells was 26.35 µM, while for RAJI (B7-H3^-^) cells, IC_50_ was >100 μM, a 3-fold selectivity or more. In conclusion, these data clearly demonstrate that Aff-MAG2 has a potent and highly specific inhibitory effect against B7-H3-expressing AML cells.

### 3.5 Aff-MAG2 induces necrotic and apoptotic cell death in AML cells

Given the encouraging viability reduction observed in the MTS assay, we sought to further characterize the functional impact of the cytotoxic construct on cellular fate using flow cytometry. THP-1 and RAJI cells were treated with 30 µM of the construct for 6 h. RAJI cells served as a negative control for B7-H3 expression and target specificity, while untreated cells from both lines constituted technical negative controls. Technical positive controls included 0.02% Triton X-100 (15 min) for necrosis induction and 30 µM Cisplatin (18 h) for apoptosis.

**FIGURE 5:**
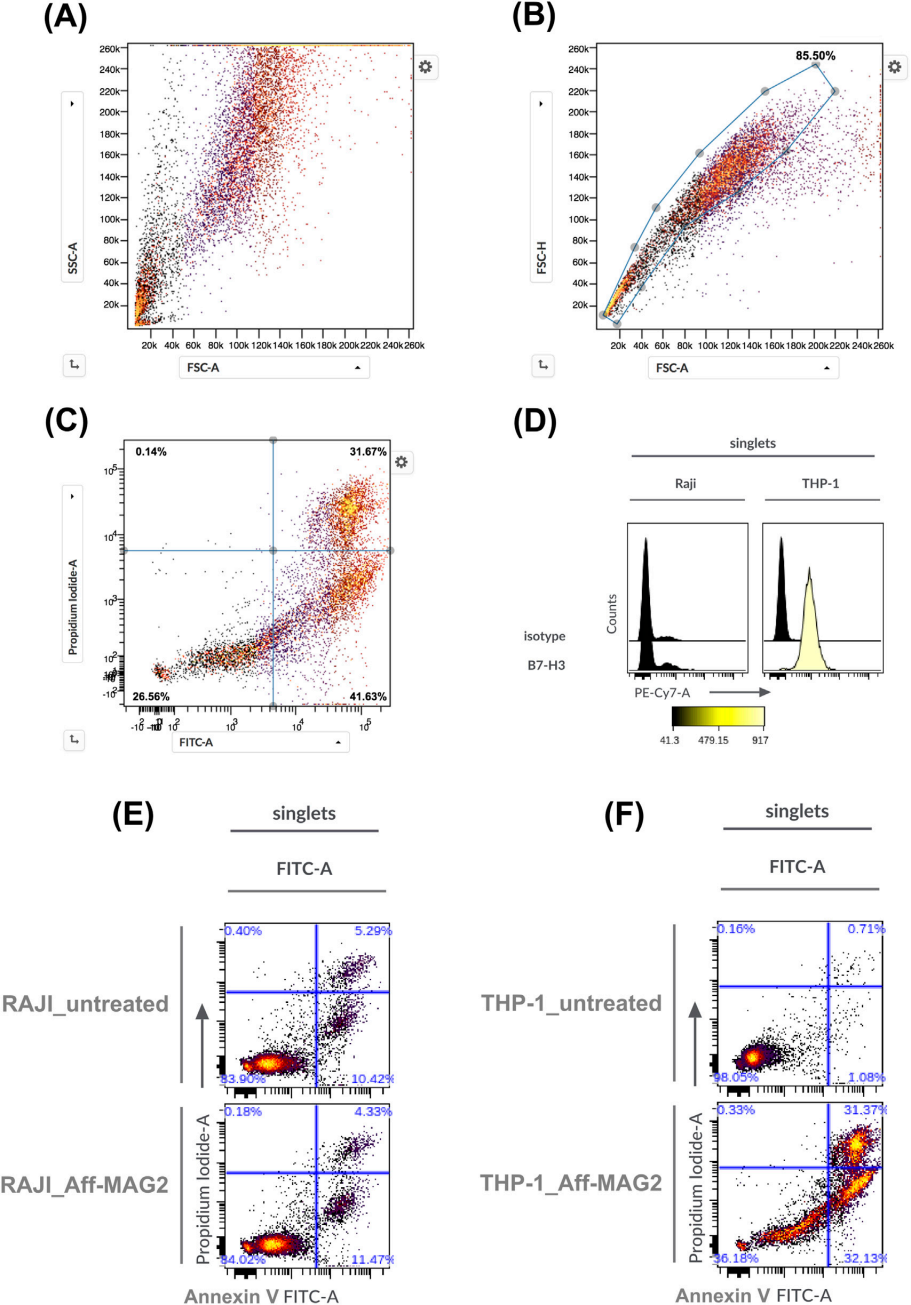
Necrosis and apoptosis of AML cells treated with Aff-MAG2, as analyzed by flow cytometry. **(A–C)** Represent the chosen gating strategies, exemplified in the case of THP-1 cells treated with the compound. In panels **(E,F)**, Aff-MAG2 - treatment with 30 µM compound for 6 h. **(A)** Ungated cells, **(B)** Singlets gate, **(C)** Cell viability, necrosis and apoptosis quadrant gate on singlet cells, **(D)** Cell surface expression of the B7-H3 antigen. On the left - the corresponding antibody used for staining, **(E)** Necrosis and apoptosis of treated vs. untreated RAJI cells, **(F)** Necrosis and apoptosis of treated vs. untreated THP-1 cells. Results from a representative flow cytometry experiment out of three performed (*n* = 3).

Figures 5A–C outlines the gating strategy used to define the cell populations of interest, while Figure 5D validates B7-H3 expression in the selected models: RAJI cells showed no detectable surface B7-H3, confirming their utility as a negative control, whereas THP-1 cells exhibited strong B7-H3 expression, validating them as an appropriate target-positive model. Supplementary Figure S3 shows the gating strategy utilized for B7-H3 expression analysis in THP-1, as a representative example.

Flow cytometry confirmed the MTS results, demonstrating a significant cytotoxic effect in THP-1 cells treated with the construct: cell viability dropped to 36.18%, with necrotic (Annexin V^+^, PI^+^) and apoptotic (Annexin V^+^, PI^−^) fractions at 31.37% and 32.13%, respectively (Figure 5F). In contrast, RAJI cell viability remained unaffected under identical treatment conditions (Figure 5E). These data corroborate the MTS findings and demonstrate that the Aff-MAG2 construct exerts a highly selective cytotoxic effect on B7-H3-expressing cells. The balanced distribution between necrotic and apoptotic subpopulations in treated THP-1 cells further supports a dual mechanism of action, with a prominent membranolytic component that can be attributed to the MAG2 peptide.

### 3.6 Aff-MAG2 decreases proliferation of AML cells

To further explore the functional consequence of Aff-MAG2 treatment, we investigated its impact on cell proliferation and apoptotic signaling. THP-1 and RAJI cells were treated with 15 µM of the compound for 6 h, followed by Western blot analysis. As before, untreated cells served as technical negative controls and RAJI cells served as the experimental negative control due to their previously described lack of B7-H3 expression.

**FIGURE 6:**
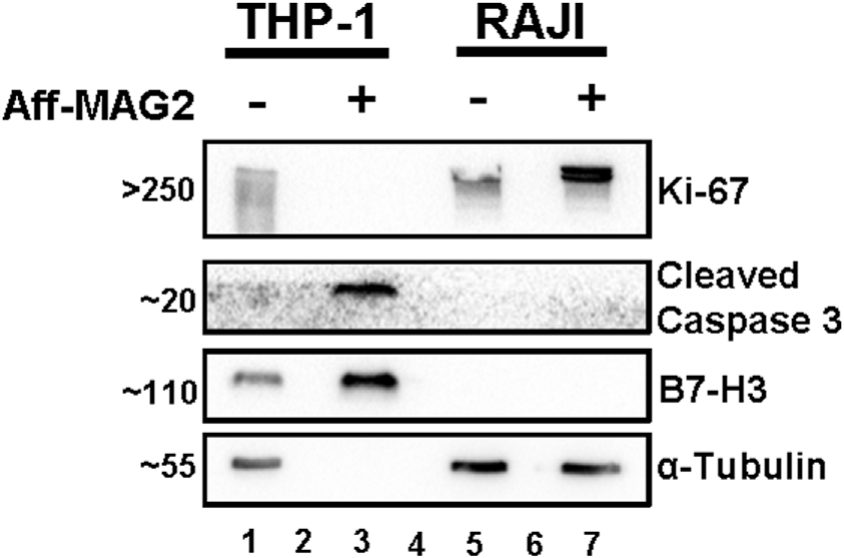
Proliferation inhibition and apoptosis increase in AML cells treated with Aff-MAG2, in Western blot. All samples were total cell lysates, loaded in equal amounts. Aff-MAG2, treatment with 15 µM cytotoxic compound for 6 h, - untreated cells, + treated cells. The numbers on the left side of the image represent the approximate mass in kDa at which the corresponding bands migrated. Lanes 2 and 6, empty; lane 4 - molecular weight marker. Probably because of extensive plasma membrane damage and loss of cytosolic content in the cell medium, the loading control α-Tubulin could not be detected in the treated THP-1 cells (lane 3). Ki-67, recognized marker for cell proliferation; Cleaved Caspase 3, recognized marker for all apoptosis pathways. Results from a representative experiment out of three performed (*n* = 3).

As shown in Figure 6, treatment with Aff-MAG2 significantly decreased the proliferation of treated THP-1 cells only, as evidenced by the complete absence of proliferation marker Ki-67 (Bading et al., 1989) in this group (lane 3). In contrast, Ki-67 remained detectable in both untreated THP-1 cells and in RAJI cells, regardless of treatment. Apoptosis was also evaluated by assessing Cleaved Caspase 3, a well-established biomarker involved in both intrinsic and extrinsic apoptotic pathways (Nicholson et al., 1995). Notably, this marker was only detected in treated THP-1 cells (lane 3), further confirming selective induction of apoptosis in B7-H3-positive cells.

We additionally validated B7-H3 expression by Western blot, observing clear bands for B7-H3 in both treated and untreated THP-1 cells (lanes 1 and 3), while RAJI cells remained negative (lanes 5 and 7).

Although α-Tubulin was used as the loading control and all samples were loaded in equal amounts, the loading control could not be detected in treated THP-1 cells (lane 3), most likely due to proteolytic membrane damage and loss of the cytosolic content. This loss of soluble cytoplasmic proteins was confirmed by the absence of other loading controls such as GAPDH (data not shown). Importantly, the presence of B7-H3 (a membrane-associated protein) and Cleaved Caspase 3 confirms that equivalent total protein was loaded across samples, despite partial loss of soluble components.

Taken together, these data support the conclusion that Aff-MAG2 selectively reduces proliferation and increases apoptosis of B7-H3-positive THP-1 cells, with minimal to no effect on non-target RAJI cells. These data complement the MTS and flow cytometry findings and reinforce the cytotoxic specificity and efficacy of this novel, targeted protein construct.

## 4 Discussion

Through this study, we endeavored to design, develop and evaluate *in vitro* a novel Affibody-Cytotoxin recombinant protein that can specifically kill B7-H3-overexpressing AML cells. This recombinant construct is made from an Affibody with previously proven high affinity against B7-H3 (Oroujeni et al., 2023) linked to Magainin-2, a potent cytotoxic membranolytic peptide, with proven cancer cell-killing properties (Lehmann et al., 2006). After designing the fusion protein such as to ensure a high expression rate in *E. coli*, with a high recovery of soluble protein, we proceeded to express it and then purify it through IMAC. High yields of pure Aff-MAG2 were obtained and the sequence of the purified protein was confirmed through nanoLC-MS/MS. We then tested the compound against THP-1 AML cells that overexpress B7-H3 and RAJI Burkitt lymphoma cells that lack the antigen of interest. The MTS assay revealed that the effect of Aff-MAG2 is dose-dependent and specific to THP-1 cells, while RAJI remained highly viable at all concentrations tested. We observed that RAJI cells are very sensitive to a multitude of external factors (vortexing, pipetting, centrifugation, etc.), some of which are essential parts of the experimental protocols we employed. This could explain the lower baseline viability of RAJI cells observed in the MTS assay and flow cytometry, even in untreated cells. We also demonstrated a strong apoptotic effect of the cytotoxic vehicle, again specific to AML cells, through flow cytometry and Western blot. This effect is accompanied by significant necrosis of THP-1 cells, as observed in flow cytometry. Given that the Magainin-2 moiety has been extensively documented to disrupt cellular membranes through pore formation (Lehmann et al., 2006), it is plausible that this mechanism contributes to the observed necrotic effect, although we did not directly visualize pore structures in this study. Notably, our Western blot experiments show that proliferation is also inhibited (Ki-67 was not detectable) in AML B7-H3-positive cells, but not in the negative control cells, RAJI. This is important, as B7-H3 is known to increase proliferation in tumor cells by increasing the levels of Ki-67 (Guo et al., 2025).

After purifying the recombinant protein, we observed some degradation, evident in Figure 2C. We tried to identify the cause for this and where Aff-MAG2 was cleaved. We reasoned that, because the affibody and the linker are known to be highly stable, the protein was most likely cleaved in the MAG2 region. The shift of ∼2 kDa between the two bands suggests cleavage near the N-terminus of MAG2. To investigate this, we utilized nanoLC-MS/MS and employed a semi-tryptic search against the recombinant sequence based on the identified peptides (Supplementary Table S1). Although semi-tryptic peptides can also result as an artefact during sample preparation and analysis, besides being caused by protein degradation, the two top-ranking peptides in Supplementary Table S1 (in bold), with the highest Peptide-Spectrum Match (PSM) counts and Sequest HT scores mapped to the N-terminal region of MAG2, near the disordered region of the linker. This recurrent detection suggests a potential degradation site within the construct (Supplementary Figure S4). We aim to explore this further in subsequent studies. Another limitation was the difficulty in identifying a suitable Western blot loading control consistently retained by the B7-H3^+^ treated cells. Testing the construct on more resilient B7-H3-negative cell lines would also provide a stronger baseline for comparison. In addition, the stability and effects of Aff-MAG2 were not tested *in vivo*, to properly observe its potential in a setting closer to the clinical one.

Although there have been over 37 years since magainins were first discovered and isolated from *Xenopus laevis* (Zasloff, 1987), studies of their effects on tumor cells are very few and far between (Baker et al., 1993; Cruciani et al., 1991; Ohsaki et al., 1992; Lehmann et al., 2006; Icriverzi et al., 2024). Even so, when compared to the studies that do exist, Aff-MAG2 is superior in several aspects. Experiments on ascites tumors in mice (including ones derived from leukemia) show that Magainin-2 is prone to proteolytic degradation in the serum and thus its effects are drastically reduced, compared to those observed *in cellulo*. Moreover, the peptide presented some toxicity in the mice tested, probably because of off-site effects on healthy tissues (Baker et al., 1993). By design, Aff-MAG2 contains stable elements in its structure (the affibody and the linker), which protect from the action of proteases. Although we have observed some degradation during purification, this likely occurs in the disordered linker/MAG2 region, as suggested by our mass spectrometry data and may be mitigated by future optimization of the purification protocol. As seen in a quantified SDS-PAGE analysis, Aff-MAG2 demonstrated a half-life of ∼24 h in cell culture medium containing serum at 37 °C (Supplementary Figure S5), supporting its stability under simulated physiological conditions. More importantly, the compound is active, highly effective and it performs as expected. Furthermore, if the degraded fraction indeed lacks MAG2, it might competitively block B7-H3 binding, potentially underestimating the maximal efficacy of the intact construct. This is because the inactive compound could possibly still block B7-H3 sites on the surface of THP-1 cells, thus competing with the functional molecules. This hypothesis will require further testing.

The IC_50_ of 26.35 µM against THP-1 cells that we hereby report is almost 3-fold smaller than the 75.2 µM reported against bladder cancer cells for simple MAG2 (Lehmann et al., 2006) and more than 2-fold smaller than the >60 µM reported by Cruciani et al., in 1991 (Cruciani et al., 1991) on several hematopoietic tumor cell lines, also using only the cytotoxic peptide. To our knowledge, the only study that obtained lower IC_50_ values than ours (∼8 µM), done by Ohsaki et al. on lung cancer cell lines (Ohsaki et al., 1992), utilized synthetic analogues of MAG2 in a 4-day treatment, compared to our 6-h treatment. Thus, it is possible that the longer incubation time could have increased the toxic effect on the treated cells. More importantly, in the same study, the IC_50_ on normal human fibroblasts was approximately between 21 and 29 µM for these analogues. This implies a much more aggressive off-site effect on normal cells, with a smaller difference from the threshold for activity on tumoral cells, than for our compound (IC_50_ for RAJI >100 µM). Although RAJI cells are also tumoral, their resistance to Aff-MAG2 suggests a favorable selectivity window for B7-H3-positive cells compared to previously reported data on non-selective toxicity of MAG2 analogues on fibroblasts. Therefore, our targeted cytotoxic molecule is much more specific to B7-H3^+^ AML cells and with considerably less collateral effects than the toxin alone, or its derivatives. Also, from what we know, this is one of the very few studies that test the Magainin-2 toxin on AML cells, conferring novelty to the approached disease as well.

It is known that in normal conditions, the high positive charge of Magainin-2 and other similar toxic peptides creates an attraction to plasma membranes, as they are negatively charged on the exterior. Even more so, it has been shown that the natural affinity of these peptides is even higher for tumor cell membranes, most likely because tumor cells expose more negatively charged residues on their surface, like phosphatidylserine (Luan et al., 2020). However, in our experiments, this affinity of MAG2 for tumoral cell membranes seems to be greatly reduced, as RAJI, although tumor cells themselves, were not significantly affected, even at the maximum tested concentration of 100 µM compound. One possible explanation for this phenomenon is a transient electrostatic interaction of the affibody with MAG2, because at physiological pH, the former has a net charge of −2, while MAG2 has a net charge of +3 (Gasteiger et al., 2005).

This potential interaction was also suggested by the AlphaFold 3 modeling of Aff-MAG2 (Figure 1B), which indicated spatial proximity and possible contacts between the affibody and the MAG2 peptide. Similar to the protective role of the SUMO tag in shielding host bacterial cells from the antibacterial effects of the toxin during its expression, the affibody might transiently interact with MAG2 to limit its non-specific activity under basal conditions. However, upon specific binding to B7-H3 on target cells, this interaction is likely disrupted or structurally altered, enabling MAG2 to exert its membranolytic effect in a localized and target-dependent manner.

The high affinity of this affibody towards B7-H3 is most likely stronger than its interaction with MAG2 and thus “frees” the toxin to attach to the cell membrane and eventually create pores in it, specifically killing only cells that highly express B7-H3. If this holds true, the construct we designed could present great potential for *in vivo* antigen-dependent activation of this potential therapeutic, warranting further testing of this hypothesis.

In future studies, we plan to stabilize Aff-MAG2 to avoid degradation, characterize its pharmacokinetic properties and its mechanism of action further through other biochemical and *in cellulo* methods, like microscopy-based approaches and test the efficacy of this novel cytotoxic construct in patient-derived xenograft AML models *in vivo*.

With the emergence of promising clinical trials targeting B7-H3-positive malignancies, such as the use of the radiolabeled monoclonal antibody (mAb) ^131^I-omburtamab on neuroblastoma patients (Kramer et al., 2022), B7-H3 has gained attention as a clinically relevant therapeutic target.

In parallel, B7-H3-directed CAR-T cells incorporating single-chain variable fragments (scFvs) against B7-H3 have shown superior efficacy over monoclonal antibodies in both solid and hematologic tumors (Guo et al., 2025), further underscoring its importance in personalized cancer therapy. In addition, targeting B7-H3 with a dual mechanism affibody-toxin fusion construct addresses the urgent need for selective AML therapies. For these reasons, we developed a novel cytotoxic molecular vehicle that is easy and cheap to produce in prokaryotes in high quantities, utilizing just simple genetic engineering without any cumbersome chemical modifications. It is highly soluble, small and does not require a delivery system to tumoral cells. Furthermore, Aff-MAG2 specifically targets AML cells that express B7-H3 with high lethal potency and little to no collateral damage, making it a promising candidate for future personalized therapy in AML and other B7-H3-positive malignancies.

## Data Availability

The datasets presented in this study can be found in online repositories. The names of the repository/repositories and accession number(s) can be found in the article/Supplementary Material.

## Abbreviations

AML, Acute Myeloid Leukemia; MAG2, Magainin-2 natural toxic peptide from *Xenopus laevis*; AfDC, Affibody-Drug Conjugate; Aff-MAG2, Anti-B7-H3 affibody coupled with Magainin-2; SUMO, Small Ubiquitin-related MOdifier; IC_50_, Half-maximal Inhibitory Concentration.
