# Supplementary material for: A novel cytotoxic anti-B7-H3 affibody with therapeutic potential in acute myeloid leukemia
Source: Front Pharmacol. 2025 Sep 12;16:1684226. doi: 10.3389/fphar.2025.1684226 (PMC12464454; doi:10.3389/fphar.2025.1684226)
Supplement: Supplementary file 1 [file DataSheet1.docx]

Supplementary Material

**1** **Supplementary Figures and Tables**

**1.1 Supplementary Figures**

**Supplementary Figure S1.** Plasmid expression vector pET-19b map produced in SnapGene Viewer. The 6xHis-SUMO-Aff-MAG2 fusion protein was inserted between the unique restriction sites NcoI and XhoI.


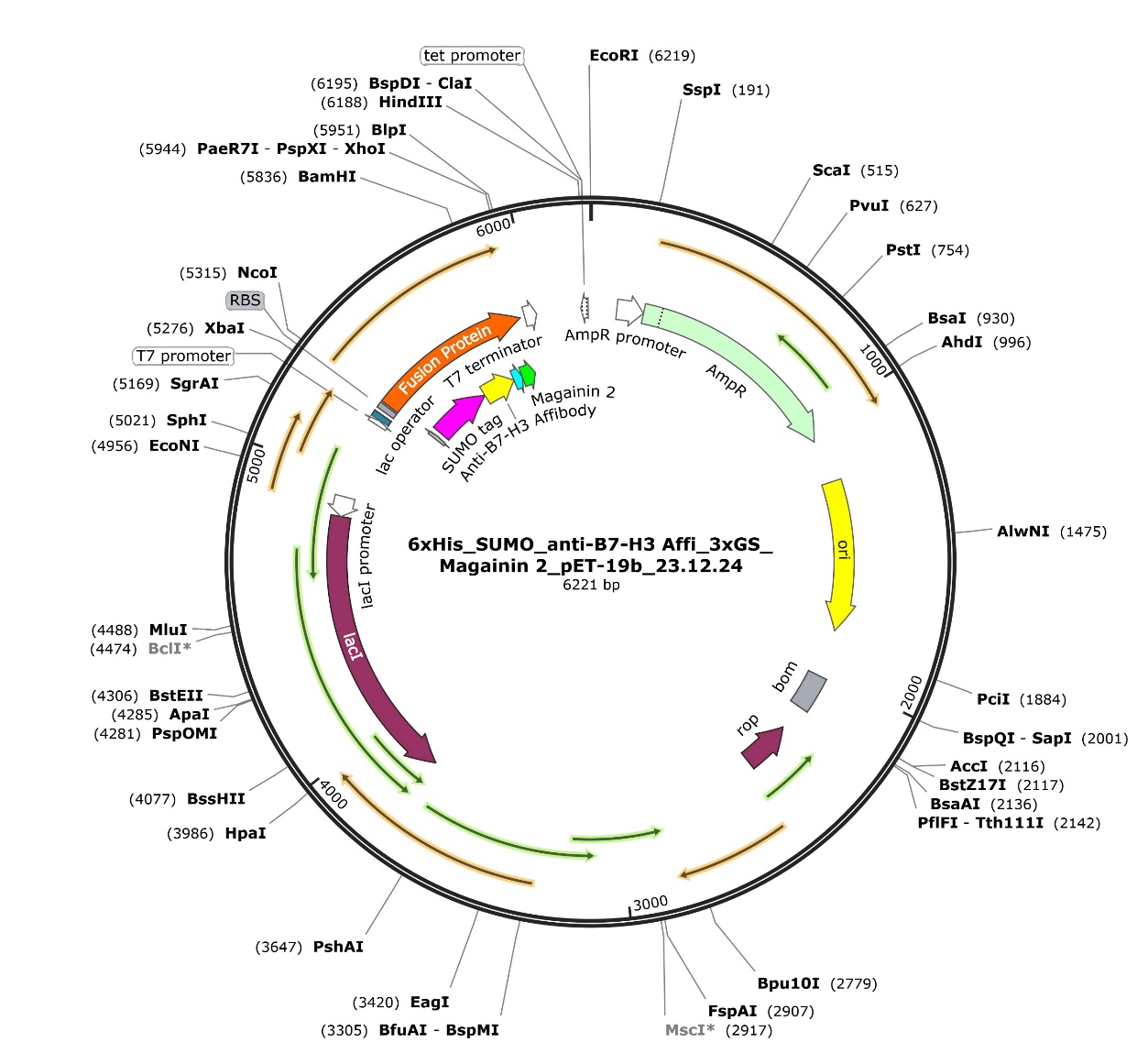


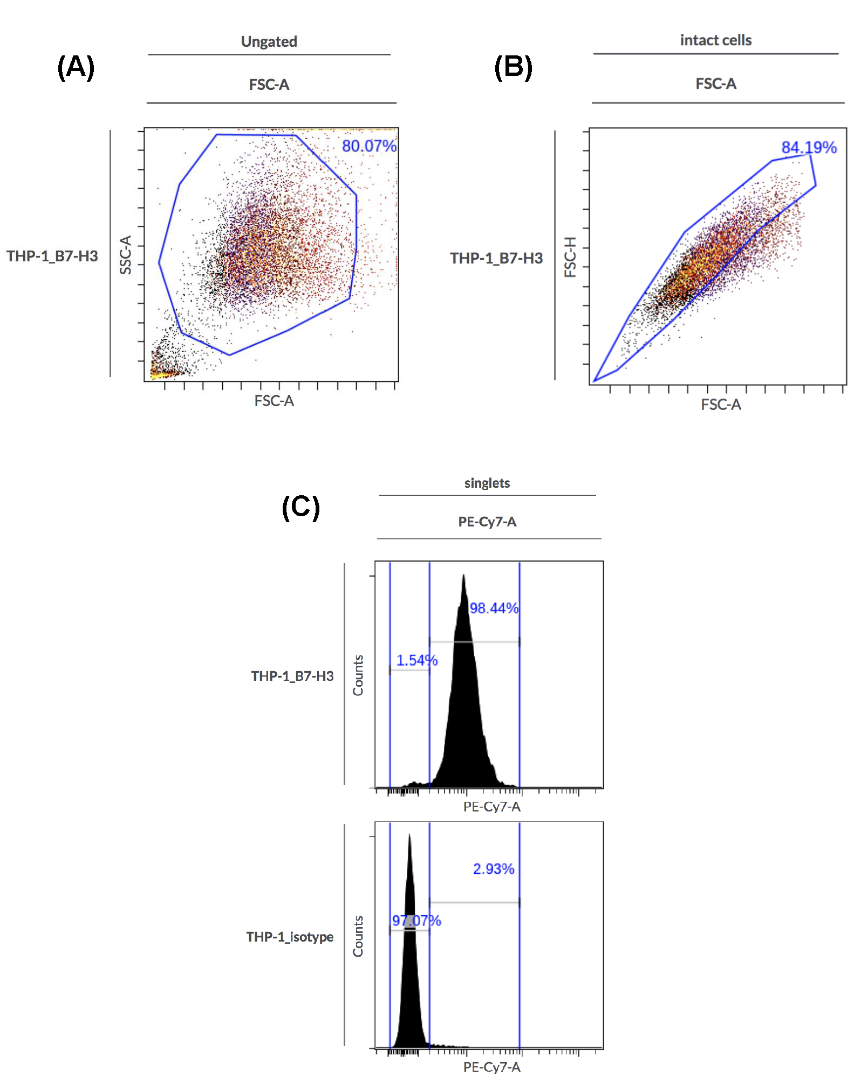
**
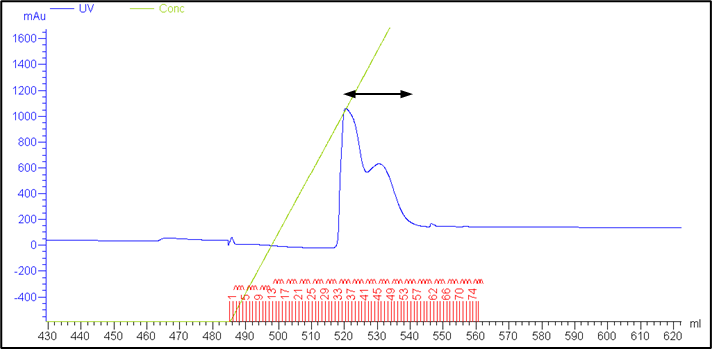
**

**Supplementary Figure S2.** FPLC chromatogram of 6xHis-SUMO-Aff-MAG2 affinity purification on HisTrap HP. The arrow indicates the 6xHis-SUMO-Aff-MAG2 elution peak. The fraction numbers correspond to the numbers of fraction samples loaded in the SDS-PAGE gel for confirmation of purification.

**
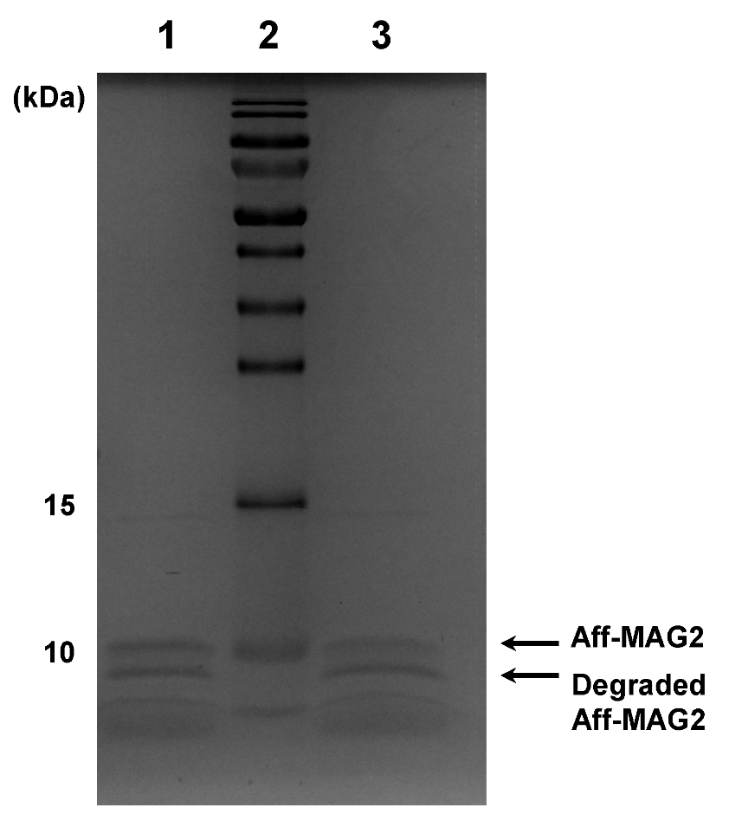

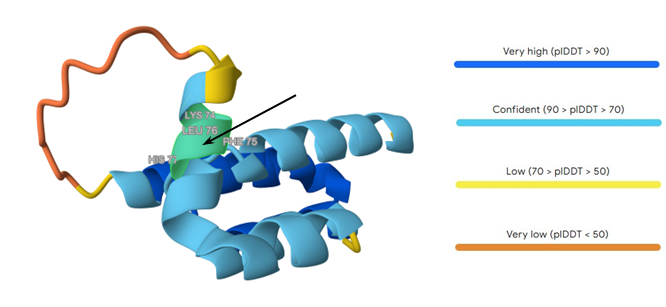
**

**Supplementary Figure S4.** 3-D structure of Aff-MAG2 simulated in AlphaFold 3 Server. The hypothetical degradation region is marked in green and the arrow indicates the probable cleavage site. The amino acids flanking the hypothetical degradation cleavage site in the vicinity of the disordered region are annotated. plDDT - predicted local Distance Difference Test.

**Supplementary Figure S3.** Representative example of the gating strategy employed for analysis of B7-H3 surface expression in THP-1 cells through flow cytometry. **(A)** FSC-A *vs.* SSC-A gating for intact cells; **(B)** FSC-A *vs.* FSC-H singlets gating; **(C)** B7-H3 positive/negative histogram gate based on PE-Cy7 fluorescence, split into anti-B7-H3 antibody and isotype control-labeled cells, respectively.

**Supplementary Figure S5.** Stability of Aff-MAG2 over time at 37 ºC in SDS-PAGE. Purified Aff-MAG2 was resuspended in the previously described cell culture medium supplemented with FBS and incubated for 24 hours at 37 ºC. **1** – purified stock Aff-MAG2; **2** – molecular weight marker; **3** – Aff-MAG2 in cell culture medium with serum after incubation. The black arrows indicate full-length Aff-MAG2 and degraded Aff-MAG2, respectively.

**1.2 Supplementary Tables**

**Supplementary Table S1.** Selected semi-tryptic peptides from mass spectrometry bottom-up analysis of purified Aff-MAG2. The most probable representative peptides for the degradation cleavage of the recombinant protein are evidenced in bold.

| **Sequence** | **# PSMs** | **Master Protein Accessions** | **# Missed Cleavages** | **Theo. MH+ [Da]** | **XCorr (by Search Engine): Sequest HT** |
| --- | --- | --- | --- | --- | --- |
| **KLSESQGGGGSGGGGSGGGGSGIGKF** | 62 | PartialMatch_Immunoglobulin | 2 | 2139.00611 | 10 |
| **LSESQGGGGSGGGGSGGGGSGIGKF** | 38 | PartialMatch_Immunoglobulin | 1 | 2010.91115 | 8.3 |
| KLSESQGGGGSGGGGSGGGGSGI | 2 | PartialMatch_Immunoglobulin | 1 | 1806.82127 | 7.38 |
| KLSESQGGGGSGGGGSGGGGSGIG | 6 | PartialMatch_Immunoglobulin | 1 | 1863.84274 | 7.36 |
| LSESQGGGGSGGGGSGGGGSGIG | 6 | PartialMatch_Immunoglobulin | 0 | 1735.74777 | 7.08 |
| LSESQGGGGSGGGGSGGGGSG | 4 | PartialMatch_Immunoglobulin | 0 | 1565.64225 | 5.99 |
| LSESQGGGGSGGGGSGGGGSGI | 2 | PartialMatch_Immunoglobulin | 0 | 1678.72631 | 5.22 |

**Supplementary Table S2.** Main buffers utilized in the study and their composition.

| **Buffer Name** | **Composition** |
| --- | --- |
| TE Buffer | 10 mM Tris, pH 8.5, 0.1 mM EDTA |
| Bacterial Lysis Buffer | 50 mM Tris, pH 8.0, 300 mM NaCl, 30 mM imidazole, 1 mM phenylmethylsulfonyl fluoride (PMSF), cOmplete™, EDTA-free Protease Inhibitor Cocktail (Roche, cat. no. 04693132001) |
| Binding Buffer | 50 mM Tris, pH 8.0, 300 mM NaCl, 30 mM imidazole |
| Elution Buffer | 50 mM Tris, pH 8.0, 300 mM NaCl, 500 mM imidazole |
| Dialysis Buffer | 50 mM Tris, pH 7.2, 300 mM NaCl, 1 mM dithiothreitol (DTT) |
| Eukaryote Lysis Buffer | RIPA buffer, Na orthovanadate 1 mM, PMSF 1 mM, cOmplete™, EDTA-free Protease Inhibitor Cocktail, iodoacetic acid 5 mM |
| FACS Buffer | 2% inactivated FBS in PBS |
| Annexin Buffer | 10 mM HEPES, pH 7.4, 2.5 mM CaCl_2_, 140 mM NaCl |
